# Policy implementation challenges and the ritualization of public health emergency plans: An investigation of urban communities in Jiangsu Province, China

**DOI:** 10.3389/fpubh.2022.1047142

**Published:** 2023-01-09

**Authors:** Rui Zhang, Chengli Wang, Changgui Li, Yachao Xiong

**Affiliations:** School of Public Policy & Management (School of Emergency Management), China University of Mining and Technology, Xuzhou, China

**Keywords:** public health emergency plans, smith policy-implementation-processing pattern, ritualization, emergencies, public health systems research

## Abstract

**Introduction:**

The COVID-19 pandemic has been a global public health emergency, and countries worldwide have responded to it through a vast array of pre-planned, adaptively devised and *ad-hoc* measures. In China, public health emergency plans - the plans expected to drive the response to epidemics or pandemics - demonstrated a concerning tendency towards “ritualization.” “Ritualization” denotes the practice of public health emergency plans to be reliably developed so that a formal requirement is met, while being implemented selectively or not at all in the emergency response.

**Methods:**

This study explored the phenomenon of ritualization by analyzing data from 1485 questionnaires, 60 in-depth interviews and 85 actual public health emergency plans. It used the Smith Policy-Implementation-Processing pattern as its conceptual framework.

**Results:**

The study found that the infeasibility of plans, their ineffective implementation by emergency management agencies, the obstructive behaviors of community residents, and the lack of an appropriate policy environment all contributed to the practice of ritualization.

**Discussion:**

As China seeks to better respond to COVID-19 and accelerate the recovery of its health system, it is essential to ensure that its public health emergency plans are effectively developed and implemented.

## 1. Introduction

The phrase “public health emergency” refers to major infectious disease outbreaks, unexplained mass diseases, primary food and occupational poisoning, and other events that seriously affect public health, which occur suddenly and cause or may cause severe damage to public health.^1^ Since 2020, the COVID-19 pandemic has been a global public health emergency that has seriously affected national economies and the livelihoods of people. Although the epidemiological situation in many countries is still not effectively controlled, an increasing number of countries are beginning to change their health policies and seek to move into a normative stage of coexistence with COVID-19. Following the 20th National Congress of the Communist Party of China, China announced that it would continue to adhere to its “dynamic zero” policy,^2^ which means that China will continue to adopt a high standard of response to COVID-19.

An emergency plan is a policy tool used in emergency management to keep the emergency response procedures on track. The effective implementation of public health emergency plans not only improves the accuracy of the emergency response, but also accelerates the recovery of a health system from the impact of COVID-19 (1). According to statistics from the Ministry of Emergency Management of the People's Republic of China, as of 2019, China has prepared more than 7.8 million emergency plans, of which more than 2 million were newly developed or revised in 2019 alone.^3^ Nonetheless, after the outbreak of COVID-19, some regions in China experienced “failures” in implementing their public health emergency plans, many of which tended to be “ritualized.”

Wang and Tang propose the concept of “ritualized law” to refer to systems that have virtually symbolic meaning and do not perform effectively in the practical application of the policy (2). In this study, “ritualization” is used to denote a pattern of behaviors concerning public health emergency planning whereby: new public health emergency plans are constantly being developed at all levels; emergency plan drills are conducted as required; emergency response subjects declare that they will activate the emergency plan during an emergency; yet emergency plans are not implemented or are selectively implemented during the emergency response. Ultimately, a public health emergency plan becomes a “decoration.” Implementing the plan becomes a “ritual,” with the plan failing to guide the practical application of a policy in the emergency response and during the recovery of the health system.

Since the outbreak of COVID-19, there has been a proliferation of research on implementing public health emergency plans. Wisniewski evaluated the effectiveness of crisis management plans (CMPs) in addressing threat risks in Poland and identified the integrity of multi-hazard plans as central to their effective implementation (3). Wolf-Fordham suggested that the development, response, and local management of emergency plans should be strengthened by promoting cooperation between emergency management departments (4). Wang et al. analyzed the implementation of public health plans in rural areas in China, classifying four types of scenarios (functional-failure, functional-delay, functional-vacancy, and functional-devaluation) in which the implementation of emergency plans fails and suggesting that “governance by law” should be vigorously strengthened (5). Li et al. analyzed the problems of community public health emergency management systems from the perspective of resilience and found that public health emergency plans at the community level were homogenized and boilerplate and that their practicality and effectiveness were poor (6). Guo and Zhao used social network analysis to study emergency plans for public health emergencies in China and found that problems such as poor linkages and generalized organizational functions made it difficult to implement them effectively (7). Overall, the existing literature suggests that features internal to a public health emergency plan and/or reflecting its fit with the environment in which it is applied, such as the plan's integrity, relevance, and pertinence, are crucial for a plan's effective implementation. Once the implementation is in progress, external factors such as legal protection, social culture, and financial support, as well as internal factors such as characteristics of the implementing organization, shape further the actual implementation of public health emergency plans. Most existing studies, however, focus on the internal factors affecting the implementation of public health emergency plans. They do not offer a comprehensive, multidimensional exploration of internal and external factors affecting a plan's implementation.

In order to explore the factors that lead to the abandonment or selective implementation of public health emergency plans, and thus help avoid their ritualization, this study proposes a framework for exploring the implementation of public health emergency plans based on Smith Policy-Implementation-Processing pattern. It represents a comprehensive model of both internal and external factors that influence the implementation of public health emergency plans. The framework informed the collection and analysis of data from 13 cities in Jiangsu province. The data were analyzed through the lens of ritualization of public health emergency plans. Recommendations are given on how to avoid the ritualization of public health emergency plans so that they can enable a more effective emergency response and contribute to an accelerated health system recovery.

## 2. Study setting and methods

### 2.1. Study setting

**Figure 1:**
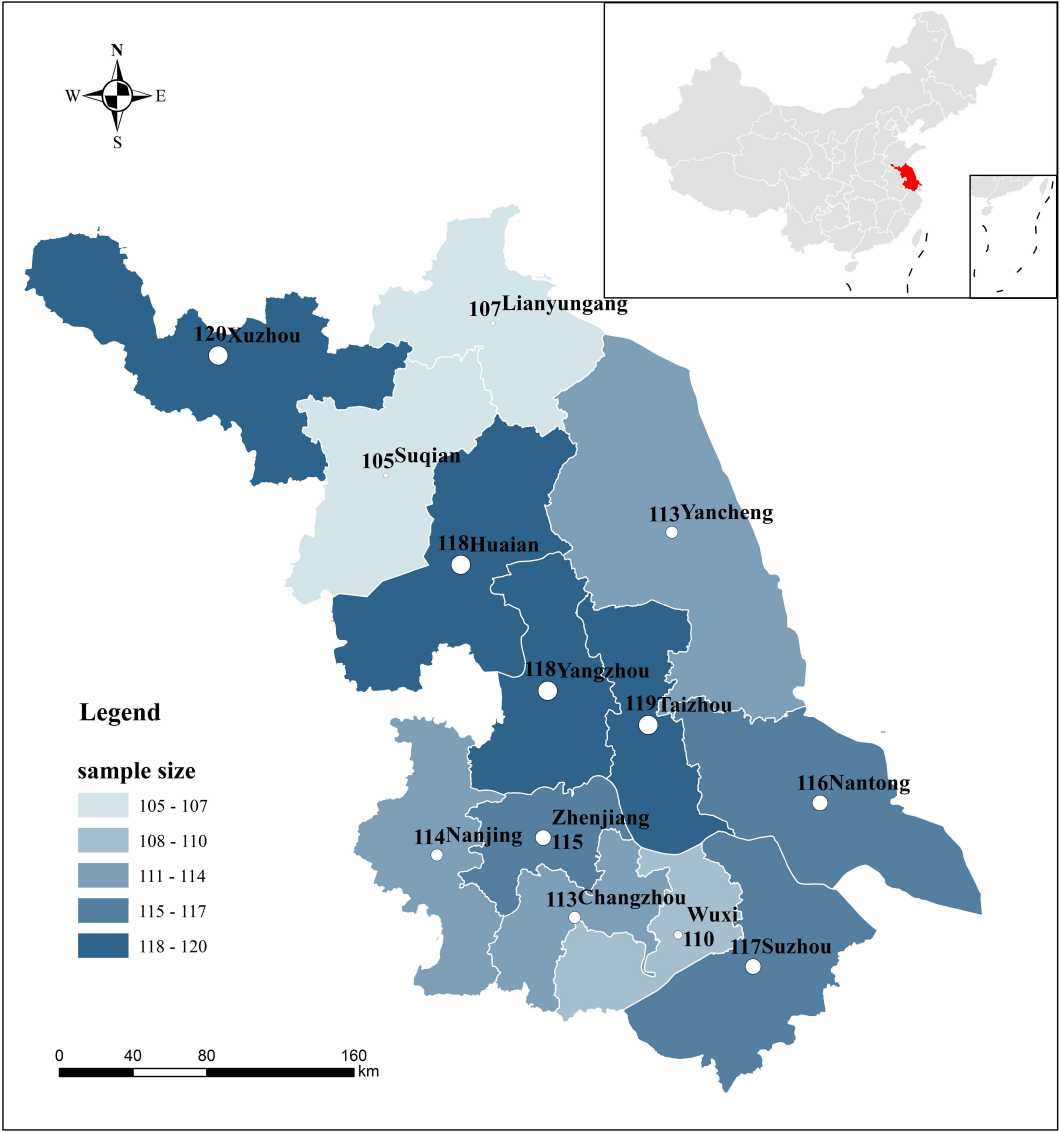
Map of the study setting (Jiangsu province and its 13 prefecture-level cities) and distribution of survey responses. The figure accompanying the name of a city represents the exact number of study participants from that city.

The study was conducted in Jiangsu Province, China. Jiangsu covers an area of 107,200 square kilometers and has 13 prefecture-level cities under its jurisdiction. By the end of 2021, the gross regional product of Jiangsu has reached 11,636.42 billion yuan, with a permanent resident population of 85 million.^4^ It is not only the province in China with the most significant population density, but also one of its most economically developed provinces. By way of comparison, Jiangsu province has a larger economy than South Korea (ranked 10th in the world) and a total population of about 30 million more than South Korea. Public health emergencies are frequent in Jiangsu due to its large and densely distributed population. In 2021, 229 public health emergencies were reported in Jiangsu (excluding the aggregated outbreak of COVID-19), with 8,490 reported cases.^5^ This makes Jiangsu an informative case study in examining the implementation of public health emergency plans in the context of COVID-19 outbreak, including the risks of their degradation into ritualized forms (location of Jiangsu Province in China and the distribution of respondents is shown in Figure 1).

### 2.2. Methods

#### 2.2.1. Smith policy-implementation-processing pattern

The Smith Policy-Implementation-Processing pattern is a theoretical model that analyzes policy implementation factors and their relationships. Smith categorized the significant factors involved in implementing a policy into four components: ideal policy, implementing organization, target group, and environmental factors (8). These four variables interact to create tensions that affect the effectiveness of policy implementation and promote or hinder the policy effects through feedback. The greatest strength of the model is its comprehensiveness and attention to tensions between the four elements.

**Figure 2:**
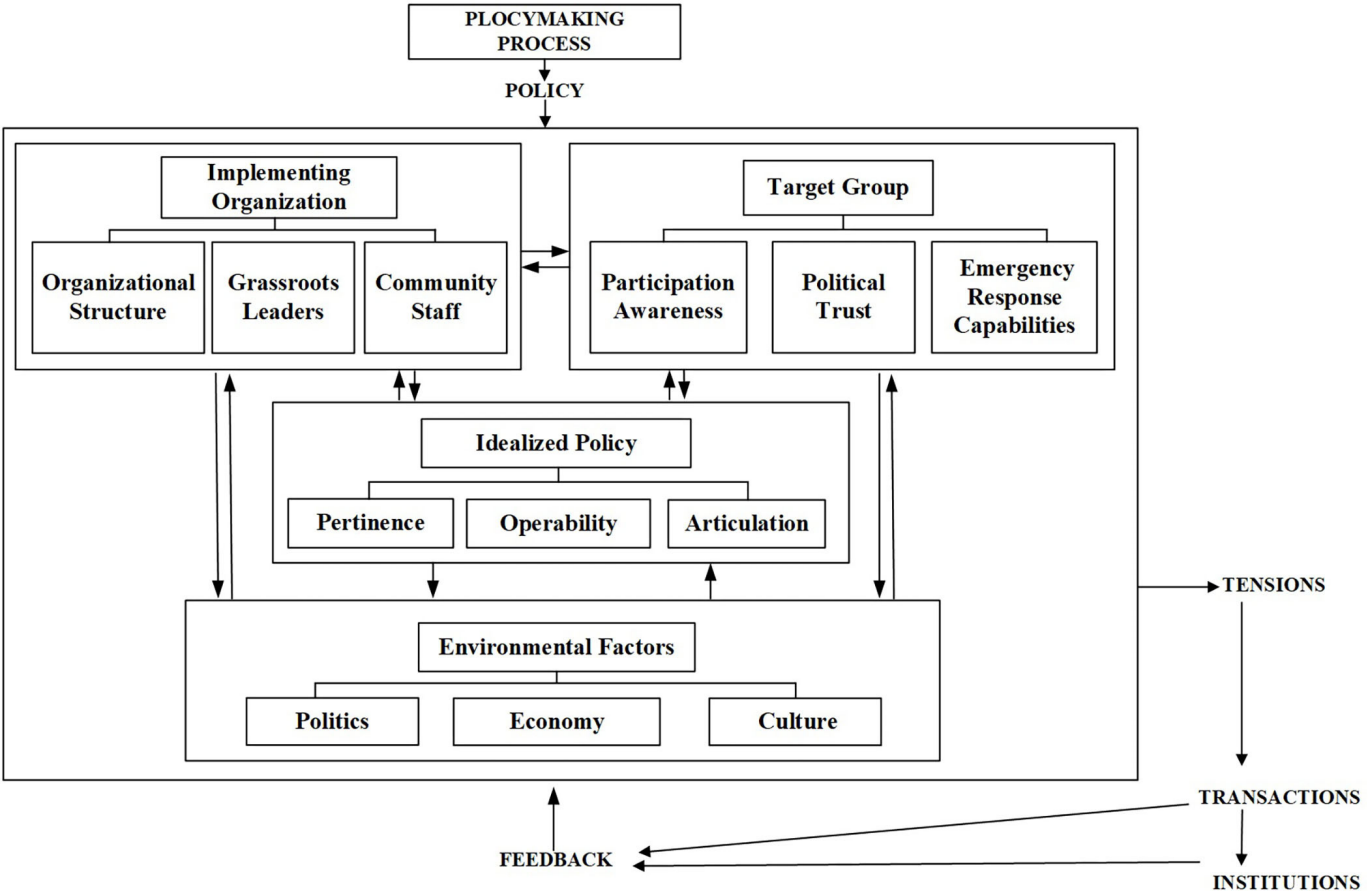
Analysis framework based on the Smith Policy-Implementation-Processing pattern.

In China, the requirements for an emergency response plan's preparation, approval, publication, exercise, evaluation, publicity and education, and organizational safeguards are specified in the Measures for the Management of Emergency Response Plans formulated by the General Office of the State Council of the People's Republic of China.^6^ The Smith Policy-Implementation-Processing pattern accounts for the variety of emergency plan management standards proposed by the Chinese government better than any alternative framework we are aware of. This study retains most of the elements of the Smith Policy-Implementation-Processing pattern, but adapts the elements included in IDEAL POLICY and TARGET GROUP. The adaptations to IDEAL POLICY concerned the features of public health emergency plans. The adaptations to TARGET GROUP concerned the group characteristics exhibited by the Chinese population (the diagram of the research framework is shown in Figure 2).

#### 2.2.2. Questionnaire

The study questionnaire was designed using constructs and their operationalization in the Smith Policy-Implementation-Processing pattern, namely the emergency plans, the implementing agents, community residents, and environmental factors (a translation of the questionnaire is available in Appendix A in Supplementary material). A total of 1,560 questionnaires were distributed, and 1,485 valid questionnaires were returned, for a response rate of 95% (details about the sampling can be found in Appendix B in Supplementary material). Researchers from China University of Mining and Technology distributed the questionnaires to respondents in face-to-face interactions. Respondents completed the questionnaires by themselves, while a researcher was available to explain items and/or response options if these were perceived to be unclear (basic information about the respondents is available in Table 1).

**Table 1:** Basic information of respondents.

**Variables**	**Options**	***N***	**Percentage (%)**
Gender^*^	Male	963	64.85
	Female	522	35.15
Age	18–29	467	31.45
	30–44	684	46.06
	45–59	319	21.48
	Over 60 years old	15	1.01
Education	Elementary school and below	5	0.34
	Junior high school	43	2.9
	High school	155	10.44
	Graduate	1,089	73.33
	Postgraduate and above	193	13

* Respondents were randomly selected, yet there was a significant difference in the proportion of male and female respondents in the final sample.

#### 2.2.3. In-depth interviews

Findings from the questionnaire were complemented by in-depth interviews with 30 community emergency staff and 30 community residents. The interviews sought rich, contextualized descriptions of the work and daily lives of respondents in relation to public health emergency plans. They allowed us to develop a more comprehensive understanding of the implementation process. Respondents were recruited during the process of questionnaire administration. If brief communications with them revealed that they could be considered “key informants,” the researcher explored their willingness to be interviewed and, if such willingness was expressed and consent given, the interview was conducted right after the questionnaire was completed. Twenty-four interviews were audio-recorded, with participants' consent. Notes were taken during the remaining interviews, as most community emergency staff preferred not to be recorded.

#### 2.2.4. Document analysis

We collected 20 emergency plans at the municipal district level and 65 community emergency plans. The analysis focused on inappropriate procedures recommended or mandated by the public health emergency plans. In particular, through comparing the textual content of plans and the actual implementation process, with the latter elucidated by questionnaire and interview findings, we could analyze the reasons for the degradation of public health emergency plans toward ritualization.

## 3. Findings

Following the analytical framework used in this study, we represent the findings in terms of the ideal policy, implementation group, target group, and policy environment.

### 3.1. Infeasibility of the public health emergency plans

Eight questionnaire items concerned the ideal policy (see Table 2).

**Table 2:** Questionnaire responses to items about the “ideal policy.”

**Item N**	**Feature of emergency plans addressed by an item**	**Yes**	**No**
		***N*** **(%)**	***N*** **(%)**
Item8	Feasibility	486 (32.7)	999 (67.3)
Item26	Effectiveness	555 (37.4)	930 (62.6)
Item9	Clear division of responsibility	591 (39.8)	894 (60.2)
Item10	Clear emergency warning process	673 (45.3)	812 (54.7)
Item11	Detailed emergency response procedures	652 (43.9)	833 (56.1)
Item12	Strong links between the different emergency plans	630 (42.4)	855 (57.6)
Item13	Consistency with higher level plans	642 (43.2)	843 (56.8)
Item14	Updated regularly	887 (59.7)	598 (40.3)

67.3% of questionnaire respondents perceived the emergency plan in their community as unfeasible. Of the 65 public health emergency plans reviewed during the document analysis stage, 27 were found to be highly similar. They copied the general measures of equivalent emergency plans, ignoring factors such as the community's geographical location, population status, and emergency supplies stocks. 60.2% of respondents felt that the distribution of primary responsibility in the public health emergency plans was unclear. 56.1% did not consider the emergency response procedures stipulated in the plan to be detailed enough. Below we provide an illustration of the vagueness of emergency response procedures, using Plan DZ from the document analysis process. The plan does not specify who would complete each step of the emergency response and how it would be done. This not only obstructs the development of an effective emergency response but may, in fact, exacerbate aspects of the emergency situation.

*DZ Community Emergency Plan (Excerpt)*

······

*Emergency response*

*1. Response procedures*

*1.1 In case of an emergency, the main person in charge of the community should immediately lead the members of the emergency leading team to the scene and quickly call the emergency team for early emergency treatment*.

*1.2 The community shall immediately report to the higher authorities and request additional emergency teams and supplies for on-site rescue*.

*1.3 The community should carry out the early rescue*.

*1.4 The community should provide information related to emergencies for the proximity of the senior leader once he or she arrives on the scene*.

*2. Aftermath response*

*2.1 The community shall stabilize the life of the people after the accident or disaster and report the implementation of the community emergency plan and the post-treatment plan*.

*2.2 The community shall be responsible for dealing with the aftermath of the dead and injured and providing compensation to their families*.

*2.3 The community shall assist the superior government and relevant departments in disaster relief work*.

*2.4 The community shall promptly undertake post-disaster recovery and reconstruction*.

*2.5 The community shall conduct disaster investigation and assessment*.

······

### 3.2. Ineffective implementation by emergency management agencies

Seven questionnaire items concerned the “implementation group” (see Table 3).

**Table 3:** Questionnaire responses to items about the “implementation group.”

**Item N**	**Feature of the emergency management agencies addressed by an item**	**Yes**	**No**
		***N*** **(%)**	***N*** **(%)**
Item15	Dedicated personnel for emergency management	1,032 (69.5)	453 (30.5)
Item16	Normative emergency management organizational structure	541 (36.4)	944 (63.6)
Item17	Implementing emergency plans according to regulations	670 (45.1)	815 (54.9)
Item18	Leaders make emergency management a priority	448 (30.2)	1,037 (69.8)
Item19	Leaders carefully check and manage emergency plans drills	527 (35.5)	958 (64.5)
Item20	Leaders prefer to take command on the spot in an emergency rather than follow the emergency plan	1,097 (73.9)	388 (26.1)
Item21	Staff have the required emergency response knowledge and skills	771 (51.9)	714 (48.1)

63.6% of the questionnaire respondents experienced the community emergency management organization as “not normative,” in the sense of not adhering to formal rules and good practices. 30.2% of respondents believed that community leaders prioritized emergency management. 35.5% of respondents believed that community leaders were strictly checking and dynamically managing public health emergency plans and drills. In the words of *Mr. Z1, a manager at Community A:*

“*All of us know that emergency plans are important, but few of us follow them in emergency response. On the one hand, leaders do not pay attention to community emergency plans, and some of them are not feasible for leaders to implement. On the other hand, we have to do our job according to what the leaders have arranged. What do we do if the leader's proximity command is different from the emergency plan? We can't go against the leaders' ideas.”*

Even when areas have a normative emergency management organization and a well-developed emergency plan, the lack of attention from community leaders can prevent emergency management work from being carried out effectively. Yet more worryingly, most community workers cannot effectively implement public health emergency plans in emergency response. 48.1% of questionnaire respondents thought that community workers did not have the expertise and skills for emergency management. Mr. S3, a staff member in charge of preparing emergency plans in B Street, reflected on the lack of professional emergency knowledge and skills of some staff members:

“*Most of my colleagues are not familiar with emergency work including emergency plans, but that can't be helped. I used to work in a related organization (Changed for anonymity), so I know a little about the preparation of earthquake emergency plan and disaster disposal, while some colleagues have no experience in emergency management before. There are prepared emergency plans in the community, but most colleagues do not have the professional knowledge and skills to implement emergency plans.”*

### 3.3. Negative impacts of target groups on the implementation of emergency plans

Six questionnaire items sought to capture the knowledge, attitudes and behaviors of the target groups (Table 4).

**Table 4:** Questionnaire responses to items about the “target group.”

**Item N**	**Features of community residents addressed by an item**	**Yes**	**No**
		***N*** **(%)**	***N*** **(%)**
Item22	Willingness to cooperate with community emergency management	472 (31.8)	1,013 (68.2)
Item23	Participated in community emergency plans drills	748 (50.4)	737 (49.6)
Item24	Willingness to actively participate in community emergency plans drills	440 (29.6)	1,045 (70.4)
Item25	Participated in the development of community emergency plans	272 (18.3)	1,213 (81.7)
Item27	Basic emergency knowledge and skills	533 (35.9)	952 (64.1)
Item28	Stocked up on basic emergency equipment or supplies at home	382 (25.7)	1,103 (74.3)

Only 31.8% of respondents were willing to participate in emergency plan drills organized by community organizations. *Ms. L1, a community resident, commented:*

“*There are few disasters here, so why waste time and money doing those drills? In my community, only when the drills or promotional activities send out gifts, everyone will be more enthusiastic about participating.”*

A significant proportion of residents lack awareness of the need to participate in emergency preparation activities and are unwilling to actively cooperate with public health emergency plan drills, which inevitably affects the community's proficiency and effectiveness in implementing plans in the emergency response. Only 35.9% of community residents believed they had basic emergency knowledge and skills.

*Ms. S2, a community resident, said, “Without COVID-19, I didn't even know we had a public health emergency plan. When it comes to emergency response capabilities, all I know is that I need to run to the open field when an earthquake happens. I don't know what emergency response knowledge and capabilities related to public health emergency plans include.”*

Community residents are the target group of public health emergency plans. Their lack of awareness of the need to participate in the emergency response and emergency management capacity limits the implementation of the plan. The emergency plan implementation group either cannot rely on the cooperation of target group members or its work is obstructed by them in the emergency response. This dramatically weakens the effectiveness of the public health emergency plan.

### 3.4. Restrictions of the policy environment on the implementation of emergency plans

Seven questionnaire items focused on the “policy environment” (Table 5).

**Table 5:** Questionnaire responses to items about the “policy environment.”

**Item N**	**Features of the policy environment addressed by an item**	**Yes**	**No**
		***N*** **(%)**	***N*** **(%)**
Item29	Political environment conducive to emergency planning	793 (53.4)	692 (46.6)
Item30	Community leaders place more emphasis on quantifiably assessable work	867 (58.4)	618 (41.6)
Item31	Community emergency management is adequately funded	499 (33.6)	986 (66.4)
Item32	The community is well stocked with emergency equipment and supplies	324 (21.8)	1,161 (78.2)
Item33	Adequate funding is available for emergency plans exercises and publicity	561 (37.8)	924 (62.2)
Item34	Participation in the development, implementation and rehearsal of community emergency plans is not necessary	931 (62.7)	554 (37.3)
Item35	Waiting passively for government assistance in emergencies	508 (34.2)	977 (65.8)

73.4% of community emergency workers felt that community leaders tended to direct on the spot rather than follow emergency plans.

In the words of *L4, a community leader:*

‘*Emergency plans are developed in response to inspections by superiors. I believe that I am successful as long as I deal with the emergency effectively, and there is no need to follow the emergency plan.”*

Regardless of whether or not the leader's knowledge, skills and proximity to the scene makes the emergency response successful, a public health emergency plan is infinitely weakened and rendered ineffective if leaders perceive its use as optional or, worse, unnecessary. This dismissive attitude was not confined to leaders. 62.7% of respondents believed that participation in developing, implementing, and rehearsing public health emergency plans was unnecessary. The sampled community residents believed that emergency management was the government's responsibility and that they did not need to be involved. In emergency response, they were more inclined to wait for rescue from the government rather than actively cooperate with the emergency response procedures. Such a social culture is, undoubtedly, a serious hindrance to the implementation of public health emergency response plans.

In terms of community emergency management resources, only 21.8% of the community emergency workers believed their communities had essential emergency equipment and reserves. 37.8% of respondents perceived the funds for emergency planning exercises and publicity as adequate. Chinese communities do not have an independent financial support system, and the funds mainly come from higher-level allocations and social donations. The lack of funds makes it difficult for communities to build disaster prevention facilities and stockpile emergency supplies.

## 4. Discussion

### 4.1. Ritualization of public health emergency plans

Our findings suggest that four main families of factors contributed to the ritualization of public health emergency plans.

Firstly, the public health emergency plan lacks pertinence, operability and articulation, which makes it unfeasible and difficult to implement. China's public health emergency plans are established forcibly based on administrative orders, and the process of plan formulation is exceptionally efficient (9). This hyper-efficiency, however, means that many of the millions of public health emergency plans are formulated quickly to cope with an inspection by a superior. These plans copy content from higher levels or other areas at the same level and tend to become the same at higher and lower levels, with no adaptation to local realities. As a result, the emergency response procedures in these plans are lacking in operability and articulation.

Second, unprofessionalism and contempt of the implementing groups leads to the abandonment or selective implementation of public health emergency plans. According to China's administrative structure, there are few civil servants at the community level dedicated to emergency management (10). Increasing the number of staff is one solution to ensure that emergency management work is undertaken by dedicated personnel. However, with more than 628,000 communities in China, every additional administrative position in a community means an enormous burden on the state. As a result, some local civil servants who do not have the knowledge and capability for emergency response are required to carry out emergency management work. In addition, in China's performance appraisal system, it is clear that community leaders place greater emphasis on quantifiable work (11). Emergency response is difficult to quantify in daily work. As a result, leaders rarely give much attention to the development, exercise, and implementation of public health emergency response plans.

Thirdly, the target group's lack of awareness of the need to participate in the emergency response and emergency response capacity increases the difficulty of implementing public health emergency plans. Activities such as emergency plan drills and self-rescue knowledge popularization need to occupy residents' non-working days. The improvement of risk prevention awareness and ability brought by these activities to residents is intangible; it cannot be quantified and cannot show immediate benefits (12). Community residents need greater awareness and motivation to participate in community emergency response activities. Indeed, some community residents do not have the knowledge and skills to participate in emergency response, even if they are willing to do so. China's previous civic education has focused more on ideological content, such as political participation, and less on emergency response skills (13). This education pattern has resulted in a lack of emergency response knowledge and skills among the general public.

Finally, the policy environment limits the implementation of public health emergency plans. Chinese community is a society of acquaintances with relatively frequent interactions and rich sharing norms, among which the most important is “Li.” According to Fei's definition, “Li is a socially accepted and appropriate code of conduct.” The so-called “Li” is “the obedience to traditional rules (14).” “Li” requires that the logic of action in dealing with affairs be carried out following the traditional established path. As an informal rule in Chinese society, “Li” obviously conflicts with the formal system. For policy executors, the implicit pursuit of the “rule of man”, which had been applied and refined in Chinese feudal society for thousands of years, makes them naturally reject implementing the established action plan. For the public, they are naturally dependent on “centralization” and tend to wait for the government to make decisions on all public matters. This has led to a tendency for emergency managers to take command on the spot during emergencies and for community residents to refrain from actively cooperating in the implementation of public health emergency plans.

### 4.2. Comparison with existing literature

This study validates some of the findings of previous studies of public health emergency plans, while also offering a more multidimensional analytical perspective. We found that the lack of feasibility was a key barrier to the effective implementation of public health emergency plans. Li proposed that China's public health emergency plan system needs to be improved and dynamically revised to connect public health emergency plans and departmental emergency plans at all levels (15). Wei et al. found that China's public health emergency plans for libraries need to be improved in relevance and operability (16). In the aftermath of SARS, Tam et al. described the success of the Canadian Pandemic Influenza Program (CPIP), which illustrated the importance of including specific, feasible, and professional emergency response procedures in emergency response plans (17). The findings of these studies are consistent with those of our study.

We found that the ineffective implementation by emergency management agencies drove the degradation of public health emergency response plans toward ritualization. Han and Zhou, in an analysis of China's public health emergency management system, found that a lack of interdepartmental collaboration combined with staff without professional competence limited the functioning of the public health system (18). Lin and Jiang, who analyzed China's public health emergency system from the perspective of safety redundancy, found that China's “pyramidal” section structure leads to poor information transfer and less efficient decision-making in emergency response (19). Changyun and Huichen provided a comprehensive review of China's emergency management capabilities during the COVID-19 pandemic and suggested that emergency management awareness and capabilities should be enhanced among local leaders (20). In contrast, this study found that the inattention of community leaders hindered the implementation of public health emergency plans. This lack of attention by community leaders stems from the political environment in China and is influenced by the cultural environment.

Several scholars have already demonstrated the importance of individuals' emergency response capabilities for emergency response. In a study of 1,252 rural residents of Jiangsu province, Zhang found that people's lack of self-rescue knowledge affects the efficiency of emergency response, and that mobilization by the Chinese government is more likely to increase the emergency response capacity of the population than advocacy by social organizations (21). Yang and Wang proposed to improve the emergency response capacity of the public by strengthening publicity and education, enhancing drills and training, and establishing cooperation mechanisms, thereby enhancing the effectiveness of the government's emergency response (22). The present study offered further insights into how target group factors and socio-cultural factors interact and impact on the implementation of public health emergency plans.

The impact of the policy environment on policy implementation has been extensively explored in classic public policy studies. However, China has a feudal history of several thousand years and a GDP growth rate of about 10% in recent decades.^7^ Its long-standing feudal culture and rapidly growing social wealth have created a unique political, economic, and cultural environment. This study provides a more comprehensive analysis of the environmental factors that have a subtle impact on the implementation of public health emergency plans than other studies. In addition, this study illustrates the influence of “Li” in Chinese society and culture. “Li” is well-suited to explain the counter-normative behavior of implementing and target groups.

### 4.3. Implications for policy, practice, and research

The ritualization issues highlighted in the COVID-19 outbreak reveal the concerning status of public health emergency response plans. China's executive order-driven emergency response planning system is gradually deviating from its original design at the community level. The COVID-19 pandemic has been ongoing for about three years, and China's public health system is gradually recovering from the severe shock it received in the early stages of the pandemic. Then, with each wave of COVID-19, the Chinese public health system was hit once again by the failure of public health emergency plans to function. This reduced the effectiveness of the government's response to COVID-19 and severely impacted the performance of the essential public health functions of the Chinese health system. As the Chinese government announces the continuation of its “dynamic zero” policy, the public health system will be under tremendous pressure for some time. Therefore, the Chinese government should work to address the “ritualization” of public health emergency plans to accelerate the recovery of the public health system and to better respond to COVID-19.

First, Chinese laws and regulations lack legal provisions directly related to emergency plans, resulting in emergency plans not having general legally binding power in public health emergencies (23). The Chinese government should strengthen the legal support for emergency plans and attach importance to the status and role of public health emergency plans. Particular policies should be formulated to regulate the process and content of the plan development to increase the operability of the plan (24). Keeping public health emergency plans feasible will increase the government's emergency response capacity and help the public health system recover from the impact of COVID-19.

Second, in practice, governments at all levels should incorporate the training, rehearsal, maintenance, and updating of public health emergency plans into the assessment of community emergency managers and establish relevant rewards and penalties, and systems to improve the motivation of community emergency managers for emergency management (25). In daily life, particularly during early stages of health systems and socioeconomic recovery, when the memory of a public health emergency is still alive and potentially traumatic, local communities should vigorously carry out various forms of emergency publicity and popularization activities to attract community residents to participate in developing and rehearsing emergency plans. The improvement of emergency managers' and community residents' emergency response capabilities not only helps implement public health emergency plans but is also an essential support for the recovery and optimization of the public health system.

Finally, existing studies tend to focus on a single factor that affects the implementation of public health emergency plans, ignoring the interaction between multiple factors. The analytical framework constructed in this study includes internal and external factors, allowing researchers to analyze the implementation of public health emergency plans more comprehensively and providing a reference for researchers to analyze similar issues. In particular, the concepts of “ritualization” and “Li” adopted in this study provide explanatory pathways for the various non-institutionalized behaviors and the persistent problems of local governance that arise in the context of formalized systems.

### 4.4. Study strengths and limitations

This study has three main advantages in comparison to previous research. First, it adopts the concept of “ritualization” to vividly describe the state of public health emergency plans that have only a virtual symbolic meaning and do not have a policy practice function. Second, it constructed an analytical framework based on the Smith Policy-Implementation-Processing pattern, offering a more multidimensional and integrated perspective than those used by previous research. Both internal and external factors were analyzed to explain comprehensively how public health emergency plans shift toward “ritualization.” Third, the study had a large sample size (1,485 survey respondents) and adopted a multi-method approach.

While we followed principles of random selection of respondents, the final sample had rather uneven proportions of men and women (64.85% and 35.15%, respectively). There appear to have been unaccounted for factors which influenced our selection of respondents during the research process. In addition, the research sites selected for this study were all urban communities due to the practicalities of access. With over 500 million people living in rural areas in China, there are significant differences between urban and rural communities. Future research needs to compare urban and rural communities in terms of patterns of ritualization of public health emergency plans.

## 5. Conclusions

This study constructed an analytical framework based on the Smith Policy-Implementation-Processing pattern and collected data from 1,485 residents in 13 prefectural-level cities in Jiangsu Province, China. The results indicated that the infeasibility of the plans, ineffective implementation by emergency management agencies, the obstructive behaviors of community residents, and the lack of an appropriate policy environment all contributed to the “ritualization” of public health emergency plans. Public health emergency plans play an important role in emergency response and in accelerating the recovery of public health systems. If public health emergency plans are far more locally adapted, feasible, and less “decorative,” members of the public, who are still experiencing the impacts of the COVID-19 pandemic, will be more likely to trust they can return to their normal rhythms of life while staying healthy. They can also have greater confidence that the lessons of the past have been incorporated into plans for the future and that future public health emergencies will not destroy lives, livelihoods and wellbeing at the scale at which COVID-19 did.

## Data availability statement

The raw data supporting the conclusions of this article will be made available by the authors, without undue reservation.

## Ethics statement

The study was approved by the Ethics Committee of the School of Public Policy and Management of the China University of Mining and Technology (approved 6-2021). This study does not involve human or animal experiments.

## Author contributions

RZ: conceptualization and methodology. RZ, CW, CL, and YX: investigation. RZ and YX: formal analysis. RZ, CW, and CL: writing—original draft. RZ and CW: writing—review and editing. All authors have read and agreed to the published version of the manuscript. All authors contributed to the article and approved the submitted version.

## Footnotes

^1^According to the “Law of the People's Republic of China on Emergency Response”. Available online: http://www.gov.cn/ziliao/flfg/2007-08/30/content_732593.htm.

^2^ https://www.mfa.gov.cn/web/wjbz_673089/xghd_673097/202205/t20220519_10689621.shtml

^3^The data comes from the Ministry of Emergency Management of the People's Republic of China. Available online: http://www.mem.gov.cn/xw/bndt/202010/t20201014_370020.shtml.

^4^The data comes from the People's Government of Jiangsu Province, People's Republic of China. Available online: http://www.jiangsu.gov.cn/art/2022/3/3/art_34151_10363589.html.

^5^Data from the official website of Jiangsu Provincial Health and Wellness Commission. http://wjw.jiangsu.gov.cn/art/2022/4/25/art_7309_10427106.html.

^6^According to the official document of the General Office of the State Council of the People's Republic of China. http://www.gov.cn/zhengce/zhengceku/2013-11/08/content_1077.htm.

^7^Data comes from the National Bureau of Statistics of the People's Republic of China. Available online: http://www.stats.gov.cn/.
