# Supplementary material for: Policy implementation challenges and the ritualization of public health emergency plans: An investigation of urban communities in Jiangsu Province, China
Source: Front Public Health. 2023 Jan 9;10:1047142. doi: 10.3389/fpubh.2022.1047142 (PMC9868851; doi:10.3389/fpubh.2022.1047142)
Supplement: Supplementary file 1 [file Presentation_1.pdf]

Appendix A

City		District	
Street		Community	
No.		Interviewer	

Affirmation: This questionnaire is for scientific research purposes only, and we promise complete confidentiality concerning the personal information involved.

1. Gender

A. Male B. Female

2.Age

A.18-29 B.30-44 C.45-59 D.60 years and above

3. Education

A. Elementary school and below B. Junior high school C. High school C. Graduate Postgraduate and above

4. Working years

A. Within a year B. 1-3years（Excluding 3）

C. 3-5years（Including 3） D. Over 5 years

5. Residence years in your community

A. Within a year B. 1-3years（Excluding 3）

C. 3-5years（Including 3） D. Over 5 years

6. Are you a community emergency worker？

A. Yes B. No

7. Does your community have an emergency plan?

A. Yes B. No

8. Is your community's emergency plan feasible in real life?

A. Yes B. No

9. Is there a clear division of responsibility in your community's emergency plan?

A. Yes B. No

10. Is there a clear emergency alert process set out in your community's community emergency plan?

A. Yes B. No

11. Are the emergency response procedures detailed in your community's Community Emergency Plan?

A. Yes B. No

12. Are there strong links between the different emergency plans in your community?

A. Yes   B. No

13. Is there a conflict in the documents between your community's emergency plan and the emergency plan of a higher authority?

A. Yes   B. No

14. Is your community's emergency plan updated regularly?

A. Yes   B. No

15. Is there dedicated personnel in your community responsible for emergency management?

A. Yes   B. No

16. Is your community's emergency management organizational structure normative?

A. Yes   B. No

17. Does your community's current emergency management organization implement emergency plans in a normative way?

A. Yes   B. No

18. Have the leaders in your community made emergency management a priority?

A. Yes   B. No

19. Do leaders in your community scrutinize and dynamically manage the rehearsal of community emergency plans?

A. Yes   B. No

20. Do leaders in your community prefer to take command on the spot during emergencies rather than follow emergency plans?

A. Yes   B. No

21. Does the level of knowledge and skills of emergency management staff in your community meet the needs of community emergency management?

A. Yes   B. No

22. Are you willing to cooperate with emergency management in your community?

A. Yes   B. No

23. Have you ever participated in an emergency preparedness exercise organized by your community?

A. Yes   B. No

24. Are you willing to participate actively in emergency preparedness drills organized by your community?

A. Yes   B. No

25. Have you been involved in the preparation of a community emergency plan?

A. Yes B. No

26. Do you think the emergency plans in your community are effective?

A. Yes B. No

27. Do you have basic emergency knowledge and skills?

A. Yes B. No

28. Do you have basic emergency equipment or substances in your house?

A. Yes B. No

29. Do you think the political environment in your area is conducive to the implementation of community emergency plans?

A. Yes B. No

30. Do you believe that your community leaders place more emphasis on quantifiably assessable work?

A. Yes B. No

31. Has the higher government provided adequate funding for emergency management in your community?

A. Yes B. No

32. Is your community well stocked with emergency equipment and supplies?

A. Yes B. No

33. Does your community have enough funds to support emergency preparedness drills and awareness?

A. Yes B. No

34. Do you believe that community members do not need to be involved in the development, implementation and rehearsal of community emergency plans?

A. Yes B. No

35. Do you think that community members only need to be reactive and wait for government action in the emergencies?

A. Yes B. No

Appendix B

In this study, stratified four-stage unequal probability sampling is adopted for calculation under the requirement that the absolute error of the sample should not exceed 3% with a confidence of 95%, and the maximum is 0.25. The sample size should be as follows:

$$n_0 = \frac{u_\alpha^2 p(1-p)}{d^2} = 1067$$

Due to multi-stage complex sampling, the design effect DEFF is about 1.5. Considering the accuracy, cost, feasibility of investigation, the sample size is determined as 1560.
